# Interactions of proteins with heparan sulfate

**DOI:** 10.1042/EBC20230093

**Published:** 2024-12-04

**Authors:** Faizah S. Alotaibi, Marim M.R. Alsadun, Sarah A. Alsaiari, Krithika Ramakrishnan, Edwin A. Yates, David G. Fernig

**Affiliations:** 1 Department of Biochemistry, Systems and Cell Biology, Institute of Molecular, Integrative and Systems Biology, University of Liverpool, Liverpool L69 7ZB, U.K.; 2 Department of Biology, University of Tabuk, Tabuk 71491, Saudi Arabia; 3 Department of Biological Sciences, College of Science, University of Jeddah, Jeddah 21589, Saudi Arabia

**Keywords:** fibroblast growth factors, glycosaminoglycans, gradient, heparan sulphate, Protein diffusion

## Abstract

Heparan sulfate (HS) is a glycosaminoglycan, polysaccharides that are considered to have arisen in the last common unicellular ancestor of multicellular animals. In this light, the large interactome of HS and its myriad functions in relation to the regulation of cell communication are not surprising. The binding of proteins to HS determines their localisation and diffusion, essential for embryonic development and homeostasis. Following the biosynthesis of the initial heparosan polymer, the subsequent modifications comprise an established canonical pathway and a minor pathway. The more frequent former starts with *N*-deacetylation and *N*-sulfation of GlcNAc residues, the latter with C-5 epimerisation of a GlcA residue adjacent to a GlcNAc. The binding of proteins to HS is driven by ionic interactions. The multivalent effect arising from the many individual ionic bonds between a single protein and a polysaccharide chain results in a far stronger interaction than would be expected from an ion-exchange process. In many instances, upon binding, both parties undergo substantial conformational change, the resulting hydrogen and van der Waal bonds contributing significant free energy to the binding reaction. Nevertheless, ionic bonds dominate the protein–polysaccharide interaction kinetically. Together with the multivalent effect, this provides an explanation for the observed trapping of HS-binding proteins in extracellular matrix. Importantly, individual ionic bonds have been observed to be dynamic; breaking and reforming, while the protein remains bound to the polysaccharide. These considerations lead to a model for 1D diffusion of proteins in extracellular matrix on HS, involving mechanisms such as sliding, chain switching and rolling.

## Introduction

The glycosaminoglycans (GAGs), particularly the sulfated heparan sulfate (HS), have long been hypothesised as primordial features of multicellularity, by virtue of their regulation of numerous extracellular protein signalling networks in animals. Circumstantial evidence was provided by the identification of genes encoding enzymes likely to be related to GAG biosynthesis in Choanoflagellates [1], considered the extant unicellular descendants of the last unicellular common ancestor of multicellular organisms [2]. The demonstration of chondroitin 6-sulfate (another GAG) in the choanoflagellate *Salpingoeca rosetta* and its regulation of sexual reproduction provided biochemical evidence for this contention [3]. Interestingly, and as noted, this function is recapitulated in mammals by a further GAG, hyaluronic acid (HA), in the cumulus oocyte coat [4]. The probable existence of at least one GAG in the unicellular ancestor of animals involved in regulating the crucial and demanding cell communication required for sexual reproduction would then facilitate, by chance, multicellular evolution.

The GAG family comprises (unsulfated) HA, synthesized and extruded through the plasma membrane, and the sulfated polysaccharides keratan sulfate, chondroitin sulfate (CS), dermatan sulfate (DS), and HS, all synthesized in the Golgi on core proteins, and subsequently directed to secretory granules, the plasma membrane or, secreted into the extracellular space to reside in extracellular matrix (ECM). While all GAGs possess extensive networks of interacting proteins, that of HS is by far the most complex. Building on early, piecemeal experiments, recent proteomics analyses have catalogued >800 extracellular HS-binding proteins [1,5–7]. These protein–HS interactions are characteristic of many regulatory circuits that underpin multicellularity. There is considerable overlap in the HS structures produced by distinct cell types, required to engage common subsets of protein ligands and, indeed, often some latitude regarding which HS sequences will bind a given protein, although binding does not always equate with activity (reviewed [8]). Owing to the central position held by HS in manifold vital metabolic control mechanisms, many pathogens also exploit HS as a cornerstone of their initial interactions with host cells (reviewed [9]), often with relatively low specificity.

## Biosynthesis of HS dictates its structure

Heparan sulfate is a secondary gene product, thus, while its biosynthetic proteins are encoded by the genome, their expression and activities are regulated by cell physiology and biochemistry to determine the structures produced. The biosynthetic proteins include sulfate transporters [10], cytosolic bifunctional 3′-phosphoadenosine 5′-phosphosulfate synthases (PAPSS) (which produces PAPS, the universal sulfate donor), Golgi transporters for PAPS and UDP-sugars (reviewed [11]), proteoglycan core proteins as well as the enzymes directly involved in the polymerisation and modification of the polysaccharide chain. By the turn of the Century, a canonical biosynthetic pathway had been mapped [12] and all the proteins involved, including those with multiple isoforms, identified [13].

**Figure 1:**
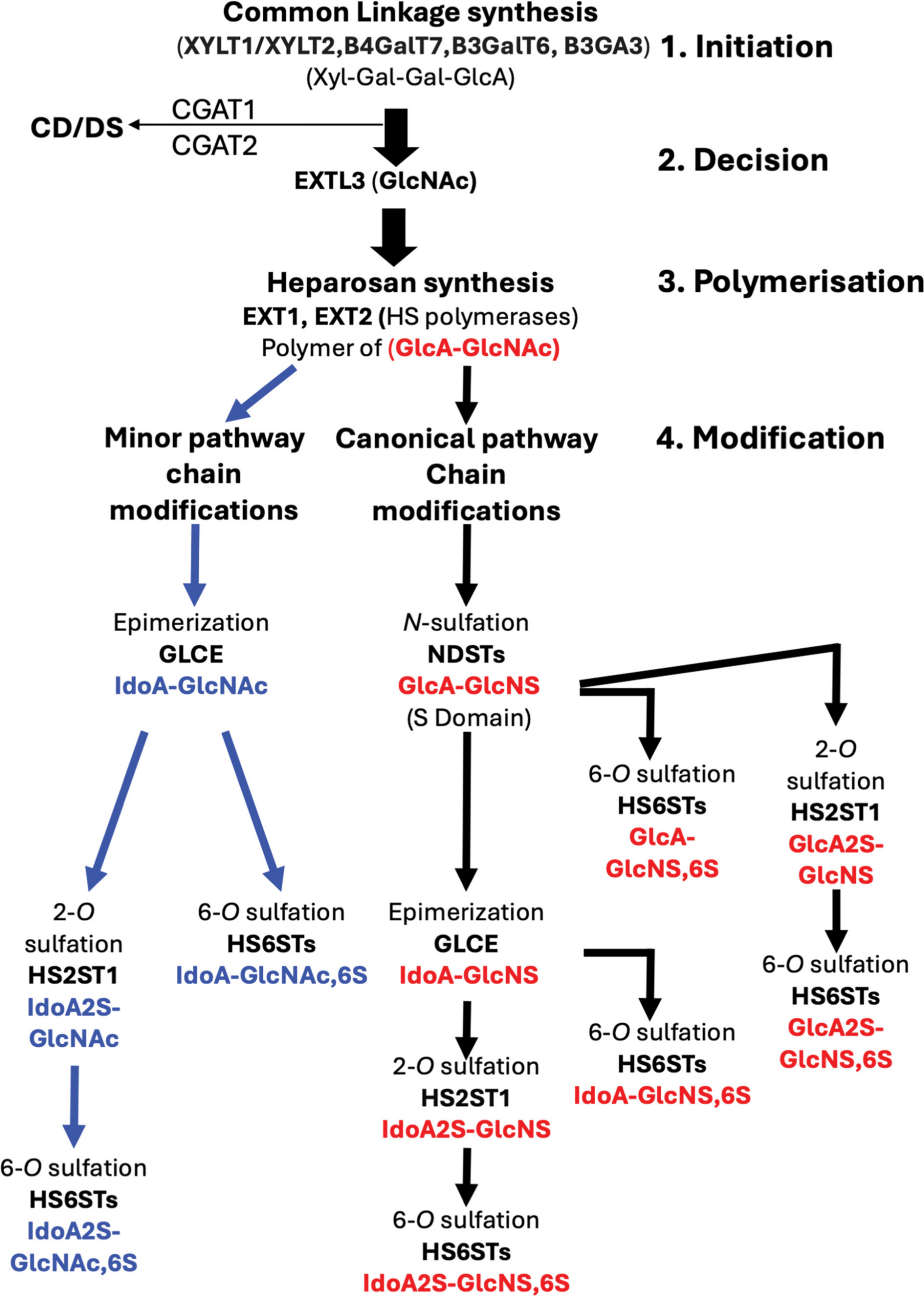
Schematic of the HS biosynthetic pathway The synthesis of the linker (initiation) to that of heparosan (polymerisation) are shown with thick black arrows. The decision step following linker synthesis is where transfer of a GalNAc by CGAT1/2 to the tetrasaccharide linker leads to CS/DS biosynthesis and of a GlcNAc by EXTL3 to HS. The modification step is in two parts. Black arrows with red disaccharides denote the canonical pathway, blue arrows and blue disaccharides the minor pathway. Enzyme abbreviations are according to UniProt.

The HS biosynthetic pathway is summarised in Figure 1. Briefly, there are four stages (Figure 1). Initiation, in which a linker tetrasaccharide, common to CS/DS and HS, is synthesised on a serine of a core protein, followed by a ‘decision’ step, at which HS and CS/DS biosynthesis diverge; addition of D-GlcNAc by EXTL3 commits to HS production, that of D-GalNAc, to CS/DS. Polymerisation involves alternating addition of glucuronate (GlcA) and GlcNAc residues by EXT1 and EXT2 and is followed by a complex set of modifications (Figure 1), most of which involve sulfation by HS sulfotransferases (HSSTs, reviewed [14]). In mammals, all the enzymes involved in the modification of the heparosan polymer except HS2ST1 and GLCE, the C5-epimerase, exist in multiple isoforms; comprising four NDSTs, three HS6STs and seven HS3STs [13]. Additional modification outside of the cell involves removal of 6-*O* sulfated from glucosamine by SULFs (reviewed [15]). The canonical pathway starts by the action of bifunctional *N*-deacetylase/*N*-sulfotransferases (NDSTs), with removal of the *N*-acetyl group from GlcNAc and its replacement with *N*-sulfate (-SO_3_^−^) (Figure 1 black arrows, red disaccharides). In the canonical pathway, all subsequent modifications perforce occur in the presence of an *N*-sulfate in the substrate disaccharide (Figure 1). The HS2ST1 has a marked preference for IdoA [16,17] and is inhibited by the presence of a C6 sulfate on the neighbouring glucosamine residue [17], which results in an ordering of these sulfation reactions. Sulfation on C3-OH of the glucosamine by HS3STs (not shown in Figure 1) is considered to occur last, and infrequently [18].

The canonical pathway does not explain the occurrence of all the experimentally observed disaccharide structures in HS isolated from animal tissues. However, it was not until 2012 that an alternative model for HS biosynthesis involving a tree structure was posited, which included a minor pathway [19] (Figure 1, blue arrows and disaccharides). Thus, bifurcation occurs at the first modification of heparosan by NDSTs acting on (-GlcA-GlcNS-) to initiate the canonical pathway, or less efficiently, by the epimerase GLCE on (-GlcA-GlcNAc-) to commence the minor pathway.

Our knowledge of HS chain structure is based on di- and oligosaccharide analysis involving degradation by bacterial heparinases, K5 lyase and nitrous acid [20] as well as extensive NMR data, for example [21,22]. At a larger scale, HS chains generally comprise sulfated (S) domains, in which every glucosamine is *N*-sulfated, flanked by transition (NAS) domains; one-in-two to one-in-three disaccharides being *N*-sulfated, the remainder, *N*-acetylated. These regions are interspersed by stretches of *N*-acetylated disaccharides (NA domains). The protein partners of HS are thought to engage the S- and NAS domains, since protein binding is dependent largely on sulfation, although sparsely sulfated regions of HS may play important biological roles and do possess negative charges on their uronic acids enabling protein binding. An important point regarding the domain structure is its generality; analyses of HS chains from particular tissues demonstrate considerable variation in domain disposition. HS from cultured fibroblasts has been shown to consist of units of NAS-S-NAS domains separated by NA domains, and there is considerable variation in the length of all domains [20]. In contrast, hepatocyte syndecan-2 (fibroglycan in the original paper) has few sulfated disaccharides in the reducing end two thirds of the chain and the non-reducing third consists largely of NAS and S domains [23].

## Interactions underpinning protein-HS binding

Proteins bind to the S and NAS domains in HS, synthesised by the canonical pathway (Figure 1), which are the most anionic structures in biology. The initial molecular approach is dominated by manifold electrostatic interactions. Charge–charge interactions between negative sulfates, carboxylates and the positive lysine and arginine side chains of proteins dominate kinetically. These are amplified by the multivalent effect consequent on multiple ionic bonds (discussed below). From an energetic standpoint, however, they may not dominate, because once the binding partners are in close proximity, further mechanisms including H-bonding, van der Waal and coordination bonding, combined with shape complementarity and the entropic effects of solvent and cation reorganisation, become increasingly important. Together, these factors provide the degree of selectivity observed for what would otherwise be an ion-exchange process.

The energetics of these ion-exchange interactions has been investigated in pioneering work using tripeptides KWK and RWR [24]; each basic group on the peptides displacing one counter ion from heparin. This entropically favourable release of counter ions, which applies equally to the surface of proteins, is the polyelectrolyte effect and is a major contributor to the free energy, Δ*G*, of the interaction of heparin/HS and proteins [24]. Direct contributions from H-bonding may be considerable between the amides of asparagine, glutamine and the backbone peptides with polar groups on HS. The bulk of the non-ionic interactions, however, occur through conformational change in the protein, induced by binding to the polysaccharide.

Conformational change in proteins induced by HS binding is a general theme, demonstrated for numerous proteins, including the paracrine a [25–27], antithrombin III [28] and the Spike protein of SARS-CoV-2 [29–31]. In many cases the conformational change in the protein is considered important to function, e.g., antithrombin III [32] and SARS-CoV-2 spike protein [29–31] but, otherwise, either no evidence is presented or, has been argued as unnecessary, as in formation of the FGF receptor ternary complex of FGF ligand, HS and FGF receptor [33].

In the well-studied case of FGF2, for which isothermal titration calorimetry showed a substantial (70%) contribution to binding free energy from H-bonding and hydrophobic interactions, the protein undergoes considerable conformational change upon interaction with heparin. This was demonstrated, for example, by the greatly increased thermal stability of FGF2 (≥30°C) for the HS bound form. The apparent contradiction with X-ray studies, which showed near superimposable Cα traces for the sugar-bound and unbound FGF2 [34] is likely to be explained by an equilibrium in solution between a more rigid structure prone to crystallisation, favoured by heparin binding, and a less rigid and thermally stable one favoured in the absence of polysaccharide.

The binding *in vitro* of FGF2 to both Cu (II) ions and heparin was demonstrated to be an effective purification strategy [35], but its potential significance was long overlooked. Subsequently, the potential for cation coordination by the numerous oxygens on heparin and a series of selectively de-sulphated heparins that provide a model for sulphated structures found in HS was explored. A combination of synchrotron radiation circular dichroism and Fourier transform infrared spectroscopy demonstrated that the polysaccharide coordinates effectively all physiological cations, which is dependent on the sulfation pattern of the polysaccharide, and that this alters the conformation and activity of the latter in solution, for example [36]. Coordination of the Cu (II) ion has been identified by virtue of the paramagnetic Cu (II) ion using electron paramagnetic resonance and NMR spectroscopy [37]. De-sulphated heparins, specifically coordinated to particular cations, e.g., a partially de-*N*-sulfated and re-*N-*acetylated heparin derivative in the presence of Cu(II) ions, exhibited altered abilities to engage in the FGF-FGF receptor-HS ternary complex and to stimulate cell division [38]. These latter experiments also demonstrate that a significant proportion of the coordinated cations remain bound, and do not exchange with the cations present in cell culture medium.

## Consequences of ionic interactions and the multivalent effect

Electrostatic interactions provide long-range steering between interacting charged macromolecular partners; the attractive forces between opposing charges providing for the re-alignment of molecules, involving conformational changes in both partners. In protein–protein interactions where the binding interface does not involve ionic bonds, electrostatic interactions outside this interface increase the rate of formation of encounter complexes, which may then convert into the bound complex or dissociate [39,40], with random molecular collisions underling the formation of the encounter complexes. Protein–HS interactions differ from these protein–protein interactions, where electrostatic interactions are part of the binding interface. Some basic residues lie outside this interface, e.g., [26,27,41,42], and similar to protein–protein interactions these may also participate in electrostatic steering. The situation is complicated in instances where there are several independent binding sites for HS on the protein surface, as identified in members of the FGF family [26,27,41,42]. Moreover, unlike a protein surface, that of the polysaccharide is far more flexible. Indeed, model HS polymers are demonstrated to occupy conformational spaces that depend on the position and number of substituted sulfates [36]. Thus, protein–protein interactions may only provide a partial model for the relationship between the encounter complex, electrostatic steering, and the final complex in a protein–HS interaction. However, these considerations, along with the multivalent effect described above may aid in understanding protein diffusion in extracellular matrices, as discussed below.

While corresponding to ion-exchange processes from the energy perspective, HS-protein interactions exhibit much stronger binding than would be anticipated on this basis alone. For example, elution of FGF2 from a sulfonyl ion exchange column requires ∼0.6 M NaCl, but needs ≥1.3 M NaCl for elution from heparin affinity columns [43]. This can be explained by the cooperative effect provided by the multivalent interactions between multiple charged species on the complementary, and conformationally plastic, polysaccharide and protein molecular scaffolds, making both molecular dissociation (c.f. individual ionic bonds) much less likely, and inhibition or competition by monovalent inhibitors, much less efficient. Thus, the latter process requires much higher electrolyte concentrations than to compete simple ion-exchange processes. Other consequences are the high association rate constant (*k*_a_) of protein–HS interactions (∼10^5^ to 10^6^ M^−1^s^−1^, e.g. [26]), and the difficulty of measuring dissociation rate constants caused by rapid rebinding. In general, if a competing oligosaccharide is not present, so that the multivalent effect prevents dissociated protein from diffusing efficiently into the bulk solution, then values for the dissociation rate constant, *k*_d_, should be treated with caution and the higher published values taken as being most likely.

Ionic interactions underpin important functional aspects of protein–HS interactions, first illustrated by the ‘trapping’ of FGF2 and other HS-binding proteins in the pericellular and ECM of cultured cells, which was ascribed to the strong ionic component of the interaction [44]. Thus, FGF2 bound to a pericellular or ECM did not dissociate into the culture medium but could, nevertheless, form ternary signalling complexes with HS and its plasma membrane receptor tyrosine kinase. Since formation of the signalling complexes occurred over hours, FGF2, while trapped on HS, could still move within the matrix. By analogy with protein–nucleic acid interactions and of the inactivation of thrombin (factor IIa) and antithrombin III by heparin, it was argued that HS-bound growth factors would move by a combination of sliding and hopping on HS chains [45].

## Transport of HS-binding proteins

### Diffusion of proteins on polyanions

There is less information available concerning the mechanisms of diffusion of proteins bound to HS but, protein–DNA interactions may provide an analogy. Protein binding is kinetically driven by ionic interactions with the polyanionic DNA, involving phosphates on the DNA backbone and basic residues on the protein. However, this binding is unable to provide differentiable features in terms of the information carried by the DNA, hence, cannot provide specificity in the functional, biological sense. To achieve the latter requires identification of particular bases within the DNA code, accessible through binding to exocyclic groups in the major groove, but such interactions do not rely on charge–charge interactions. *In vitro* experiments and molecular dynamics demonstrate that small DNA-binding proteins can both hop and slide [46], the extent of each motion relating to the number of basic residues on the protein surface that engage the DNA; sliding requiring five or more. These motions limit diffusion to one-dimension, the importance of which has been demonstrated elegantly using single molecule analysis of *lac* repressor binding to the *Escherichia coli* chromosome [47].

In contrast, in the case of protein-HS binding, while interactions also rely strongly on charge-charge interactions, this time with the negatively charged sulfate and carboxylate groups that are appended to the exterior of the HS chain, it is the varied spatial and geometric disposition of these groups that enables their recognition by complementary positively charged protein surfaces and imparts selectivity and specificity. One of the challenges facing research into HS is the difficulty of obtaining sufficient pure and homogenous material to enable interpretation of such interaction studies, since HS is a polydisperse polymer comprising a complex mixture of different dimensions and sequences. Unlike DNA, there is no readily accessible amplification system by which single molecular species, especially of polysaccharide dimensions, can be produced. Thus, the study of protein–DNA interactions provides inspiration, but the lessons drawn are not transposable to protein–HS interactions.

### Evidence for the regulation of protein transport by HS

The regulation of the location and the movement of proteins in the extracellular space, whether very short (molecular dimensions) or much longer range (organ dimensions) is critical to multicellularity. HS is likely to be the regulator of the localisation and movement of the >800 extracellular proteins to which it binds [5,6] and is central to controlling information transfer between cells and to generating instructive gradients.

The interaction of heparin with antithrombin III (ATIII) has been studied in great depth. It requires sequences of saccharides capable of promoting, it has been variously proposed, either a conformational change [48], or, alternatively, of providing stabilisation [49]. In any case, the most effective oligosaccharides isolated from heparin contain the 3-*O* sulfated disaccharide [50,51] in a pentasaccharide sequence [52] upon which the synthetic anticoagulant Arixtra™ is based [53]. Thrombin, in contrast, exhibits more relaxed selectivity [54] and, once bound, is able to slide along the sugar chain. The inactivation of thrombin by heparin can be accompanied by both the activation of AT and a reduction of the encounter between antithrombin and thrombin to that of 1D diffusion, thereby significantly catalysing effective encounters.

There are numerous examples of the role of HS–protein interactions in regulating the transport between cells of morphogens, growth factors, cytokines and chemokines. One is the diffusion of hedgehog protein, in the development of the *Drosophila melanogaster* wing and eye discs [55,56]. Another example from among many morphogens, growth factors, cytokines and chemokines, is vascular endothelial growth factor (VEGF), synthesized in multiple isoforms with varied HS-binding properties, varying from none (VEGF_121_) to strong binding (VEGF_188_). Genetic manipulation of the isoforms produced demonstrates unequivocally that the intermediate level of HS binding possessed by the VEGF_165_ isoform is essential for source cells to generate a VEGF gradient for effective angiogenesis [57].

FGF diffusion has been analysed by single and ensemble microscopy. Single molecule microscopy revealed that FGF2 trapped in the pericellular matrix of fibroblasts was unable to dissociate into the bulk culture medium yet retained full mobility. Different classes of diffusion were identified and individual FGF2 molecules were observed to sample these randomly. Intriguingly, electron microscopy demonstrated that FGF2 proteins were clustered over distances from 10 nm to 1 µm [58]. Fluorescence recovery after photobleaching of four different FGFs in the same fibroblasts again demonstrated trapping in the ECM, as well as diffusion within it by FGFs1, 2 and 6 although, interestingly, not by FGF10, which remained stationary at these length and time scales [59]. This was consistent with the demonstration that in a model of salivary gland morphogenesis, FGF10 activity depends on heparanase; heparanase being able to cleave HS chains and liberate FGF10-bound oligosaccharides [60]. Thus, while hopping and sliding is common, there are cases where a HS-binding protein is unable to move over the distances probed by optical microscopy (∼250 nm) and their movement would, in fact, require further processing of the HS or of proteins in the ECM by hydrolases.

Pathogens that bind HS are likely to be subject to the same processes identified for single host proteins. The receptor binding domain of SARS-CoV-2 binds HS, driving conformational change in the Spike protein to enable interaction with ACE-2 receptors, the prelude to viral cell entry and reproduction in the host. Many sugar-binding viruses, for example, influenza, possess both a sugar receptor binding protein and a glycosidase, HA and NA, respectively. Movement of the influenza virus within host tissue is the result of the asymmetry of the vial particle and of the distribution of NA on the particle’s surface. Together this results in asymmetric degradation of the sugar receptor, generating a local gradient of sialic acid, providing the means for HA binding to sialic acid to move the particle directionally [61]. Many HS binding viruses such as SARS-CoV-2, however, do not possess a glycosidase. This suggests that movement of SARS-CoV-2 viral particles in a tissue, necessary to successfully infect a host, must depend on the ionic interactions of the Spike protein with host HS. In this instance, several binding modes for the Spike protein and HS have been proposed, Spike protein–HS interactions are multivalent in themselves and the virus particle possesses many Spike proteins, so is also multivalent at this level [62].

### Multivalent ionic bonding provides the dynamics necessary for protein diffusion on HS in extracellular matrix

**Figure 2:**
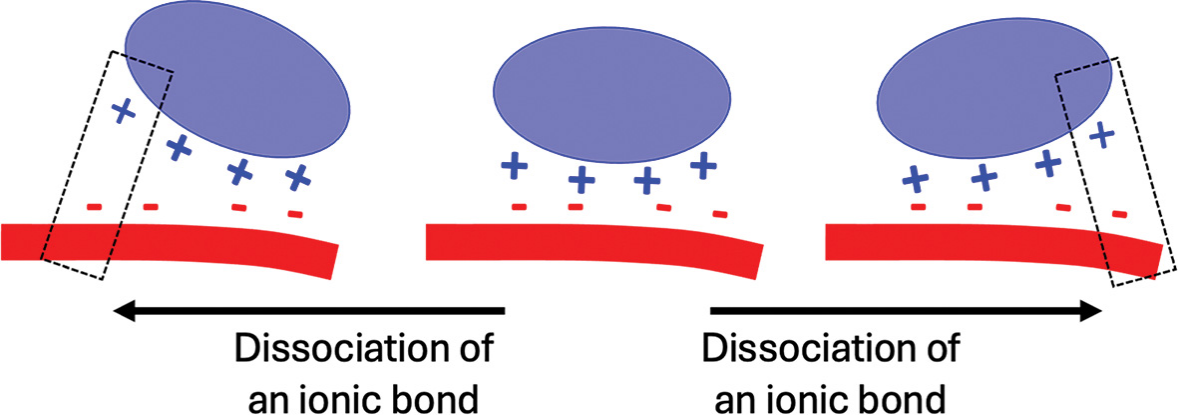
Schematic of dynamics of ionic bonds of a protein bound to an HS chain Centre, the protein has four ionic bonds with HS. Left and right, transient dissociation of ionic bonds results in the protein only binding by two of the ionic bonds.

The multivalent ionic interactions of an individual protein with a HS chain are likely to be one of the keys to understanding how HS-binding proteins diffuse in extracellular matrix with HS. A combination of different modes of movement can be envisaged. Sliding of a protein on HS requires individual ionic bonds to break and reform while, by virtue of the multivalent effect, the protein remains bound to the polysaccharide. Hopping necessitates a brief dissociation at the molecular level, which in the context of the distance over which ionic bonds are effective, may be only partial. There is extensive evidence of the mechanisms underlying the multivalent effect. The protect and label approach used to identify HS-binding lysine and arginine side chains involves initial chemical protection of their non-binding side chains that are exposed to solvent in the polysaccharide bound protein. Following dissociation of protein and polysaccharide, those side chains involved in binding are exposed to solvent through dissociation and are then labelled [41,42]. In the case of FGF1, FGF7, FGF18 [26] and FGF10 [27], some lysine and arginine side chains were demonstrated to be both protected and labelled. This can only occur if the individual ionic bonds partially dissociate in the time frame of the protection reaction – a demonstration of the mechanism underlying the multivalent effect, illustrated in Figure 2. A further example is provided by neuropilin 1 [63]. Interestingly, in the case of FGF2, elegant single molecule biophysics experiments demonstrate that the FGF2 interaction with heparin is characterised by slip bonds (non covalent bonds whose dissociation rate decreases upon action of a tensile force), which, upon mechanically pulling the FGF2 on heparin, failed to dissociate it; instead, the FGF2 moved along the polysaccharide; sequential dissociation and association of ionic bonds enabling the protein to remain bound [64].

Macromolecular crowding is another important feature of extracellular matrix. The effects of crowding have been addressed in some preliminary experiments [65]. The results demonstrate that *in vitro* crowding agents reduce the rate of diffusion of FGF2 and its apparent on-rate for an immobilised heparin tetradecasaccharide, which is of a length comparable to that of the FGF2. What might occur in the more crowded and complex environment of the extracellular matrix, where the FGF2 would be binding the considerably longer chains of HS is not known. Crowding may affect the formation and dissociation of encounter complexes, perhaps extending their lifetime. Moreover, given the multivalent effect discussed above, the dissociation rate of bound complexes may more frequently involve encounter complexes rather than full molecular dissociation. Regardless, these preliminary data suggest that molecular crowding may play a hitherto overlooked role in protein–HS interactions.

**Figure 3:**
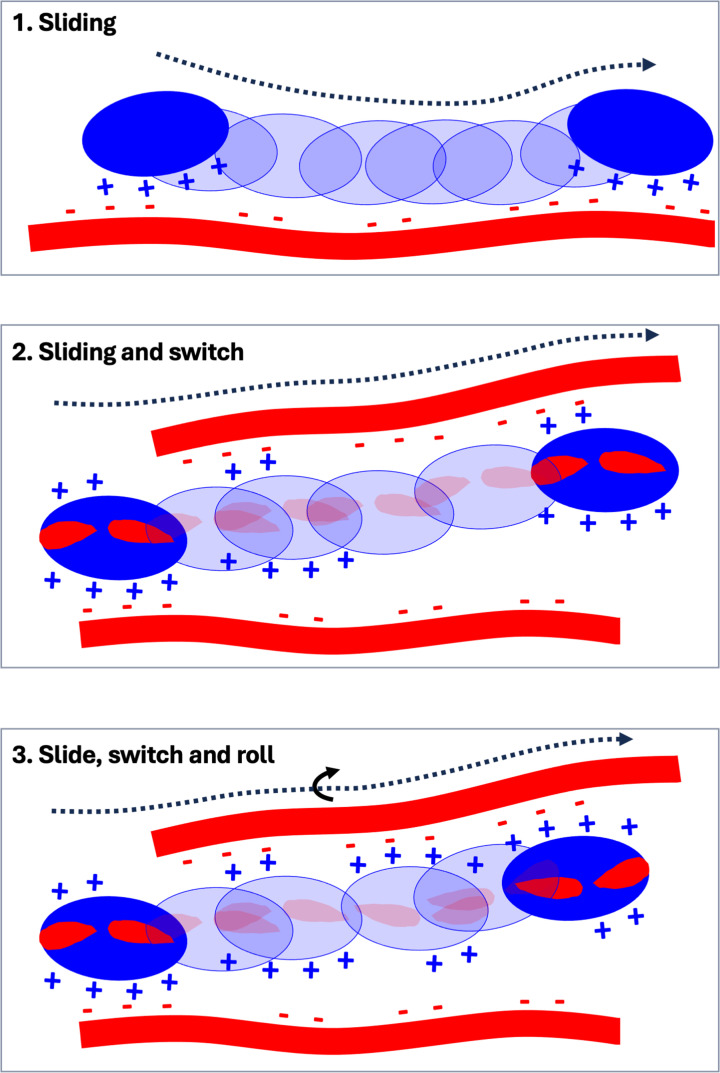
Schematic of some of the types of 1-dimensional diffusion that are proposed to underlie at least some of the movement of HS-binding proteins in extracellular matrix **Sliding** shows a protein sliding along a HS chain while remaining bound to it by virtue of transient dissociation/association of individual ionic bonds. **Sliding and switch** illustrates a HS-binding protein with two independent binding sites, isolated from each other by acidic patches (shown in red) on the protein surface. The minor binding site (two positive charges) engages negatively charged groups on a neighbouring HS chain; sliding results in the protein switching the chain with which it is associated. **Slide, switch and roll** is similar to ‘Slide and switch’ except that after switching chains, the protein undergoes a roll, such that its major binding site (four positive charges) now engages the chain rather than the minor binding site (two positive charges).

Proteins bind in preference to the more sulfated regions of HS chains (S- and NAS domains) for which their binding surface has the greatest complementarity. However, while sulfate groups are concentrated in S- and NAS-domains (Figure 1), the NA domains possess one carboxylic acid per disaccharide, and the action of the minor pathway (Figure 1) may add the occasional sulfate group to these regions. Together, the multivalent effect and macromolecular crowding may enable a HS-binding protein to slide from one higher affinity NAS-S-NAS domain to another without fully disengaging from the polysaccharide (Figure 3). These would not have to be on the same chain, since the multivalent effect would allow a protein to switch chains (Figure 3), without either fully dissociating into the surrounding medium. This propensity may also be enhanced in proteins with more than one independent HS-binding site, such as FGF2 [41,66], some other members of the FGF family [26,27,42] and of the hedgehog family [56], which could be envisioned to ‘roll’, employing different heparan sulfate binding sites (Figure 3). The consequence of these considerations is that a number of different modes of movement of a protein on HS chains can be envisaged: sliding; sliding and switching; rolling (Figure 3).

A final point to consider is that the extracellular matrix is far from uniform in composition or in the complement of sulfated structures on its HS. The latter point has been demonstrated in a number of tissues using HS-binding proteins, including 'phage display antibodies that recognise restricted subsets of sulfated structures, as observed in the lungs of developing rat embryos [67]. Indeed, the differences in gradients observed *in vivo* with VEGFs with different HS-binding affinities [51,68] could occur for a single protein species by means of extracellular matrix containing HS species of differing sulfation, so charge, and/or sulfation pattern that form a gradient in a tissue.

## Summary

- Understanding how proteins move in extracellular matrix is important in development, tissue repair and infectious diseases. In-depth biophysical analysis of the movement of proteins in HS matrices is required to establish experimentally the relationship between the protein–polysaccharide interaction and the dynamics of protein movement.
- In relation to the above point, would coordination of different cations, dependent on organism physiology and perhaps environmental exposure, affect protein movement?
- Studies of protein movement in extracellular matrix demonstrate that individual exogenously added protein species bound to HS are heterogeneously distributed. Do these proteins bind to HS structures associated with distinct, local ‘micro proteomes’ that are functionally important?
- Studies of exogenous proteins trapped in extracellular matrix have focussed on receptor ligands, but there are many other classes of HS-binding proteins. Do the endogenous HS bound proteins of extracellular matrix possess the same mobility as observed for the exogenously added ones? If so, extracellular matrix would, be far more dynamic at the molecular level than considered hitherto.

## Open Access

Open access for this article was enabled by the participation of University of Liverpool in an all-inclusive *Read & Publish* agreement with Portland Press and the Biochemical Society under a transformative agreement with JISC.

## Abbreviations

CS: chondroitin sulfate
DS: dermatan sulfate
ECM: extracellular matrix
FGF: fibroblast growth factor
GAG: glycosaminoglycan
HA: hyaluronic acid
HS: heparan sulfate
NA-domain: *N*-acetyl domain
NAS-domain: *N*-acetyl-sulfated-*N*-acetyl transition
PAPS: 3′-phosphoadenosine 5′-phosphosulfate
PAPSS: 3′-phosphoadenosine 5′-phosphosulfate synthase
S-domain: sulfated domain
VEGF: vascular endothelial growth factor
